# NOTCH and Graft-Versus-Host Disease

**DOI:** 10.3389/fimmu.2018.01825

**Published:** 2018-08-10

**Authors:** Mauro Di Ianni, Beatrice Del Papa, Stefano Baldoni, Ambra Di Tommaso, Bianca Fabi, Emanuela Rosati, Annalisa Natale, Stella Santarone, Paola Olioso, Gabriele Papalinetti, Raffaella Giancola, Patrizia Accorsi, Paolo Di Bartolomeo, Paolo Sportoletti, Franca Falzetti

**Affiliations:** ^1^Department of Medicine and Aging Sciences, University of Chieti-Pescara, Chieti, Italy; ^2^Department of Hematology, Transfusion Medicine and Biotechnologies, Ospedale Civile, Pescara, Italy; ^3^Institute of Hematology-Centro di Ricerche Emato-Oncologiche (CREO), University of Perugia, Perugia, Italy; ^4^Department of Life, Health and Environmental Sciences, Hematology Section, University of L’Aquila, L’Aquila, Italy; ^5^Department of Experimental Medicine, Biosciences and Medical Embriology Section, University of Perugia, Perugia, Italy

**Keywords:** NOTCH, graft-versus-host disease, tolerance, graft-versus-leukemia, HSCT

## Abstract

In allogeneic hematopoietic stem cell transplantation, which is the major curative therapy for hematological malignancies, T cells play a key role in the development of graft-versus-host disease (GvHD). NOTCH pathway is a conserved signal transduction system that regulates T cell development and differentiation. The present review analyses the role of the NOTCH signaling as a new regulator of acute GvHD. NOTCH signaling could also represent a new therapeutic target for GvHD.

## Introduction

Hematopoietic stem cell transplantation from allogeneic donors is the major curative therapy for hematological malignancies such as acute leukemias (ALs). The development of graft-versus-host disease (GvHD) is the most common complication which dramatically increases post-transplant morbidity and mortality (1). The clinical presentations of GvHD include acute GvHD (aGvHD) which regards 30–50% of transplanted patients and chronic GvHD (cGvHD) which includes 30–70% of patients who underwent allogeneic hematopoietic stem cell transplantation (2, 3). GvHD is triggered by the donor T cells that can cause an inflammatory disease ultimately leading to severe multiorgan damage (liver, gut, and skin) (4–8).

Donor T cells play a crucial role not only in mediating the onset of GvHD but also in eradicating malignancy, the graft-versus-leukemia (GvL) effect as showed by clinical (9, 10) and experimental studies (11–13). Allogeneic T cells recognize host antigens on leukemic cells and leukemia-specific responses may also occur (14). Despite this strong GvL effect exerted by donor T cells, relapse is still the major cause of treatment failure in high-risk AL patients who underwent allogeneic HSCT (15–18). Strategies to separate GvHD and GvL are then under investigation.

The NOTCH signaling pathway relies on the interactions between receptors (NOTCH1–4) and ligands (Jagged1 and -2 or Dll1, -3, and -4) that are expressed on neighboring cells (19). The interactions NOTCH/NOTCH ligand induce proteolytic activation of the receptor by an ADAM family metalloprotease and then by the γ-secretase complex. The sequential cuts lead to the release of the active intracellular NOTCH (ICN) that enters the nucleus and interacts with the DNA binding CSL/RBP-Jk factor, constituting a transcriptional activation complex with a mastermind-like (MAML) family coactivator. This ultimately promotes the transcription of target genes, controlling crucial biologic processes, such as survival, proliferation, and differentiation (20). Besides the canonical ICN/CSL/MAML-dependent transcriptional activation, RBP-Jk-independent non-canonical NOTCH signaling have also described (21, 22).

NOTCH signaling was first studied for its fundamental role in the early step of lymphopoiesis (23) and it has been implicated also in mature T cell function (24–26). More recently, NOTCH signaling has emerged as a new regulator of acute (27–32) and cGvHD (33). In this review, we will focus on NOTCH signaling and aGvHD.

## NOTCH Signaling is Activated During GvHD in Donor T Cells

NOTCH and alloimmune responses have been extensively studied in GvHD and in non-GvHd models. In Severe Aplastic Anemia (SAA) mouse model, Roderick et al. (34) showed NOTCH signaling mediate Th1 cell differentiation and T-BET expression. Treatment with γ-secretase inhibitors (GSIs) reduced NOTCH and T-BET expression and rescued mice from SAA.

In the setting of GvHD, the Kean group (35) demonstrated the existence of NOTCH-related signature in alloreactive T cells harvested from a non-human primate model.

The Maillard group reported that NOTCH signaling is a strong regulator of T-cell activation, differentiation, and function during GvHD (28, 36). Murine models of allo-HCT showed that inhibition of canonical NOTCH signaling markedly decreased GVHD severity and mortality (28–30). NOTCH inhibition dramatically reduced the accumulation of alloreactive T cells in the gut. Interestingly, NOTCH-inhibited T cells significantly retained their antileukemic activity. By using humanized antibodies and conditional genetic models, Tran et al. (29) demonstrated that all the effects of NOTCH signaling during GvHD were dependent on NOTCH1/2 receptors in T cells and Dll1/4 ligands in the recipient, with dominant roles for NOTCH1 and Dll4 (29). NOTCH-inhibited T cells acquire a hyporesponsive phenotype in both CD4 and CD8 populations. NOTCH deprived T cells markedly reduced cytokine production but maintain their expansion capacity and their *in vitro* cytotoxic ability (30).

The exact mechanisms of NOTCH modulation in T cells remain to be elucidated. Mochizuki et al. (37) in murine model showed that during GvHD, inflammatory DCs Dll4 ligand positive produce significantly high level of IFN-γ and IL-17. More recently, Chung et al. (27) showed that NOTCH signal are delivered to donor T cells shortly after transplantation and that host stromal cells are the source for NOTCH ligands during *in vivo* priming of alloreactive T cells. Interestingly, Luo et al. (38) have shown in an MHC-mismatched murine all-BMT model, inhibition of NOTCH signaling reduce the incidence of GvHD by reducing DCs and CD8 T cell proliferation and activation.

**Figure 1:**
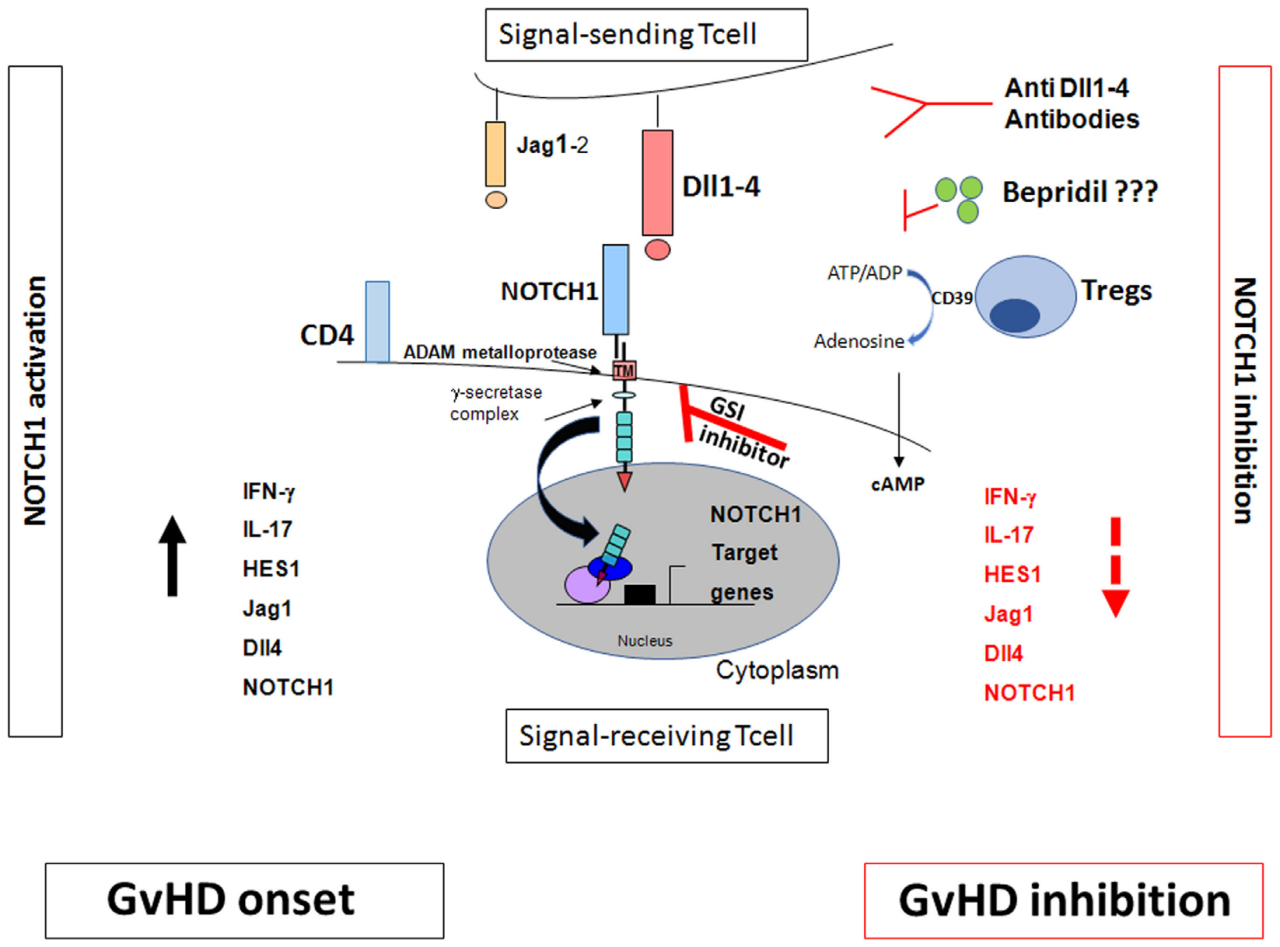
NOTCH signaling and graft-versus-host disease (GvHD). The effects of NOTCH signaling during GvHD are dependent on NOTCH1/2 receptors in T cells and Dll1/4 ligands in the recipient, with dominant roles for NOTCH1 and Dll4. Inhibition of canonical NOTCH signaling by γ-secretase inhibitor (GSI)-inhibitor, anti Dll1–4 antibodies, bepridil, and regulatory T cells (Tregs) markedly decreased GVHD (28–30). Tregs inhibit the NOTCH/NOTCH ligand interactions. They act through the CD39/adenosine axis to inhibit the NOTCH pathway which, in turn, regulates T cell proliferation and consequently inhibits GvHD. Blocking CD39/adenosine axis reverts NOTCH inhibition and favors GvHD onset (49).

NOTCH pathway inhibition could be therapeutically targeted by: (1) GSIs that block the proteolytic activation after the NOTCH/NOTCH ligand interaction (39). However, the use of GSIs in murine model of GvHD is associated with a severe toxicity in the gut epithelium (29); (2) monoclonal antibodies directed against NOTCH ligands such as Dll1–4 (29); (3) we recently identified the calcium channel modulator bepridil as a new NOTCH1 pathway inhibitor in Chronic Lymphocytic Leukemia (40). It represents an attractive therapeutic strategy to prevent also GvHD (Figure 1).

## Regulatory T Cells (Tregs) Downregulate NOTCH Signaling in Donor T Cells

Regulatory T cells suppressed alloimmune reactions like, for example, GvHD (41). They also promoted tolerance to allogeneic organ transplants (42). Adoptive Treg/conventional T cell (Tcons)-based immunotherapy in full-haplotype mismatched transplantation practically eliminated acute and cGvHD, supported post-transplant immunological reconstitution and exerted a strong GvL effect (43–48) in high-risk AL patients.

Although the mechanisms underlying Treg suppression of GvHD with no loss of GvL activity remain to be unraveled, the principal hypotheses are based on (a) the Treg/Tcon homing and distribution patterns and (b) different molecular pathways in Tcon activation and proliferation and, consequently, GvL and GvHD. Interestingly, using humanized antibodies and conditional genetic mouse models to inactivate NOTCH signaling in donor T cells markedly reduced GvHD severity and mortality (28–30). NOTCH signaling other than a cell autonomous mechanism can be modulated with an extrinsic signal from an adjacent interacting cell. Current evidence suggests that Tregs and anti-NOTCH1 compounds inhibit the same NOTCH ligands and receptors on Tcons (29, 49). Mimicking the drug-mediated NOTCH1 inhibition (30), Tregs directly inhibited NOTCH1 signaling on Tcons *in vitro* and *in vivo*, with the blockade being observed on CD4 and CD8 cells from mouse lymph nodes (49). Jagged1 and Dll4 NOTCH1 ligands, played major roles (49) with Dll4 being reported to mediate all NOTCH signaling effects in Tcons during GvHD (29). As a GvHD prevention strategy, using alloantigen-specific Tregs which preferentially inhibit alloreactive Tcons to downregulate NOTCH1 clearly offers advantages over administering pharmaceutical agents which exert a total blockade on NOTCH1 signaling on all Tcons.

CD39–NOTCH1 pathway crosstalk was also demonstrated (49). In fact, NOTCH1 expression and signaling on Tcons were restored when CD39 was blocked by the anti-CD39 monoclonal antibody or polyoxometalate-1 (POM-1), the selective CD39 inhibitor (49). Increased cAMP levels were associated with NOTCH1 reduction in Tcons; adding anti-CD39 reduced cAMP levels and reversed the Treg-mediated NOTCH1 reduction. GvHD reappeared in mice after POM-1 administration (49). *In vitro* studies (50–52) showed that blocking Abs or chemical products downmodulated the CD39/adenosine axis and reversed Treg suppression of T cons. Although the Treg mechanisms of action are multiple and partially unclear (53), these data showed that Tregs triggered NOTCH1 downregulation directly in Tcons and acted through the CD39/adenosine axis to inhibit the NOTCH pathway which, in turn, regulates Tcon proliferation (Figure 1). This mechanism of action could account for Treg-induced inhibition of Tcon proliferation which was observed by others (30).

Interestingly, in CD4 and CD8 cell populations, NOTCH1 downregulation was more marked in peripheral blood than in bone marrow (54). Tregs were demonstrated to block Tcons in the periphery but not in bone marrow (55). We could speculate that Treg homing patterns play a major role in these results. Tregs could have downregulated NOTCH1 expression in peripheral tissue because they homed there while, because of different migratory properties, they homed less efficiently, or not at all, to bone marrow. Translation of tissue-specific NOTCH1 expression into a strong GvL effect without GvHD, needs, however, to be elucidated in depth. A Treg-related NOTCH1 blockade could account for clinical and experimental evidence that Tregs prevented GvHD and facilitated a powerful Tcon-dependent GvL effect (44, 45). Consequently Treg-mediated NOTCH inhibition, like drug-induced NOTCH downregulation (28–30) may separate GvHD from GvL. This finding has major implications for adoptive immunotherapy strategies in the field of transplantation for leukemia.

## Mesenchymal Stem Cells (MSCs) Recruit Induced Tregs (iTregs) by Activating NOTCH Signaling

**Figure 2:**
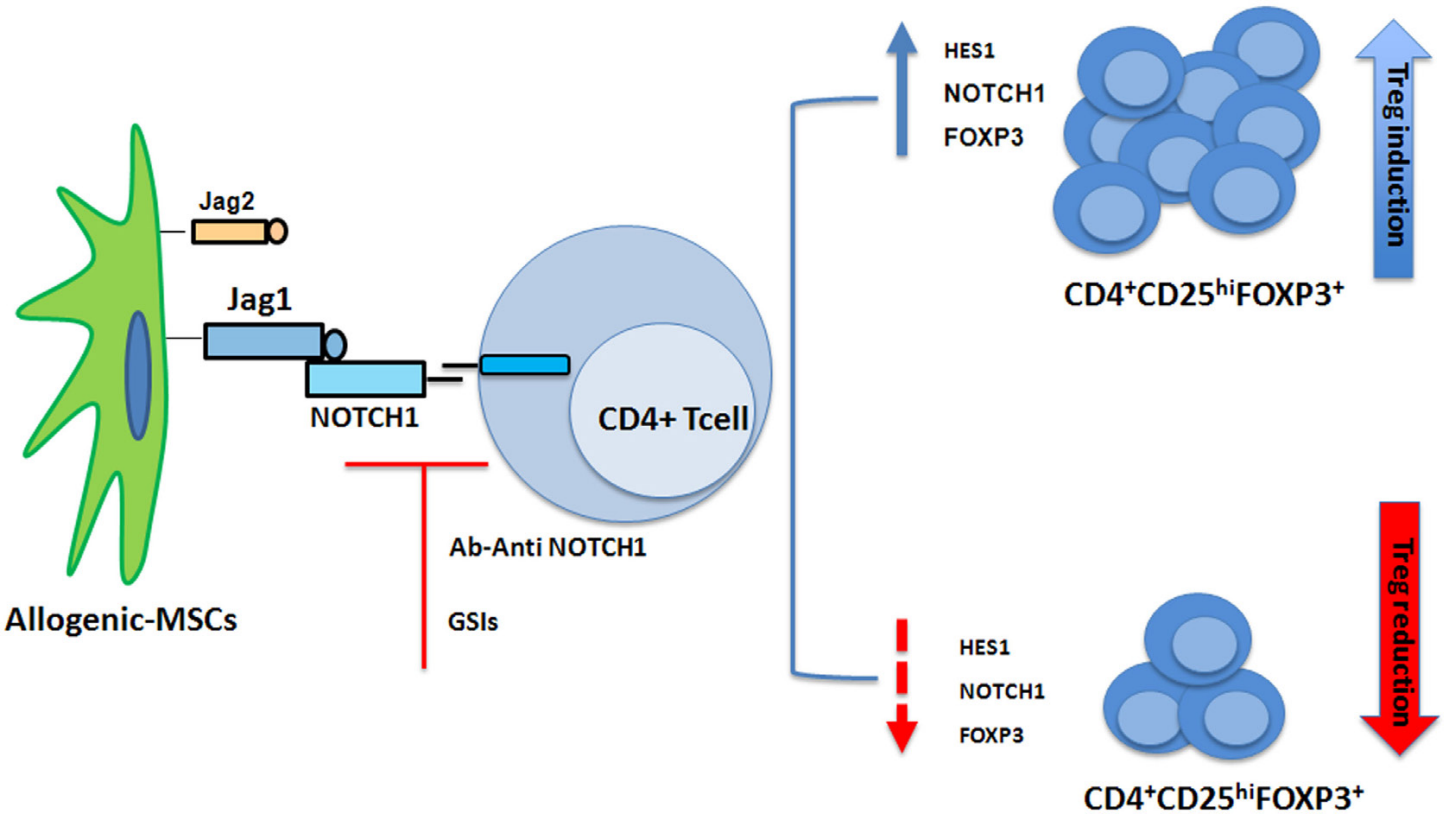
Mesenchymal stem cells (MSCs) recruit induced Tregs (iTregs) by activating Notch signaling. When cocultured with CD4+ cells, MSCs induced a T-cell population with a regulatory phenotype (iTregs) (57). NOTCH1 pathway is activated in CD4+ T cells cocultured with MSCs. Inhibition of NOTCH1 signaling through γ-secretase inhibitor (GSI)-I or the NOTCH1 neutralizing antibody reduced expression of HES1 and the percentage of MSC-induced CD4+CD25highFOXP3+ cells *in vitro* (58).

NOTCH1 signaling is also involved in Treg cell differentiation. Liotta et al. had described Jagged1 involvement in MSC suppression of T-cell proliferation (56). Our group showed when cocultured with CD3+ cells, MSCs induced a T-cell population with a regulatory phenotype (57). When CD4+ T cells were cocultured with MSCs, the NOTCH1 pathway was found to be activated (58). Using GSI-I or the NOTCH1 neutralizing antibody to inhibit NOTCH1 signaling reduced HES1 expression (the NOTCH1 downstream target) and the percentage of MSC-induced CD4+CD25highFOXP3+ cells *in vitro* (58) (Figure 2). In human cells FOXP3 is another NOTCH signaling downstream target (58), thus data from murine models were extended (59). NOTCH signaling activation reversed the unstable regulatory/suppressive properties of iTreg cells, ensuring sustained FOXP3 expression and stable Treg-cell phenotypes (58). No crosstalk between NOTCH1 and TGF-β signaling pathways was observed (58). Previous studies had demonstrated TGF-β production was involved in MSCs-mediated Treg cell induction (60, 61) and reported TGF-β/NOTCH1 crosstalk (58) in peripheral Treg cell maintenance. Lack of T-cell receptor stimulation in the work by Del Papa et al. may account for the discrepancy with other reports (58, 62–64). Together, these findings indicated that NOTCH1 pathway activation played a role in MSC-mediated human Treg-cell induction. In conclusion while on one side our observation on MSC-T cell coculture suggest a positive role of NOTCH in the generation of iTregs, on the other side NOTCH inhibition (drug or Treg mediated) in mature donor T cells is associated with reduction in GvHD severity and mortality.

## Conclusion and Perspectives

Allogeneic immune system played a crucial role not only in mediating the onset of GvHD but also in eradicating malignancy, i.e., the GvL effect. Separating GvHD from GvL represent a major challenge. GvHD prophylaxis and treatment is mainly based on immunosuppressive treatment with drugs such as cyclosporine, tacrolimus, methotrexate, antithymocyte globulin, and glucocorticoids (4). Data reviewed here showed NOTCH1 as a new major regulator of alloreactivity. Triggering NOTCH pathway with pharmacological (GSIs, Ab anti-Notch) or cellular (Tregs) ways might represent a new strategy to separate GvHD from GvL.

## Author Contributions

MDI organized the plan and structure of the manuscript, and all the authors contributed to the redaction.

## Conflict of Interest Statement

The authors declare that the research was conducted in the absence of any commercial or financial relationships that could be construed as a potential conflict of interest.
